# Expanding Global Learning Through Virtual Exchange: A COIL Pilot in Primary Care and Health Education Conducted by MSUCOM and University of Nigeria Students

**DOI:** 10.51894/001c.144148

**Published:** 2025-09-30

**Authors:** Lelti Asgedom

**Affiliations:** 1 College of Osteopathic Medicine Michigan State University

## 46

### INTRODUCTION/BACKGROUND

Global learning opportunities provide students with incredible experiences to expand not only their classroom knowledge but also attributes such as cultural awareness, sensitivity, and the ability to connect and work with individuals from different parts of the world. Some of the biggest challenges to global learning include cost and safety, as showcased by the COVID-19 pandemic. Collaborative Online International Learning (COIL) is an educational framework connecting students and faculty globally through virtual exchange. It provides a flexible format for intercultural learning, multidisciplinary applications, and collaboration. The Michigan State University Global Health Studies Program partnered with the University of Nigeria, Nsukka Department of Human Kinetics and Health Education to deliver a short pilot course on primary care and primary health care. Primary care emphasizes medical services such as disease diagnosis, treatment, and management, while primary health care takes a more community-centered approach, focusing on health promotion and disease prevention.

### AIMS/OBJECTIVES

The course objectives included identifying key attributes of primary care and primary health care, interpreting these attributes through storytelling, comparing and critiquing them, and improving intercultural communication, cultural self-awareness, perspective-taking, and critical thinking.

### METHODS

Students from both institutions participated in structured discussions and qualitative research through qualitative interviews analyzed via basic content analysis in groups. These interviews were conducted over the Zoom platform.

### RESULTS

Findings highlighted notable similarities and differences between primary care and primary health care in Nigeria and the United States. In terms of access, long wait times in public clinics were common in both settings, but Nigerian respondents faced greater travel-related challenges. Regarding quality, while providers were attentive, overcrowded public clinics led to compromised care. Continuity of care was facilitated by patient records in both countries, but the U.S. used electronic health records more widely, while Nigeria relied on manual records.

### DISCUSSION/CONCLUSIONS

Recommendations included improving accessibility through mobile healthcare units, increasing staffing, implementing electronic health records, promoting follow-up care, and expanding preventative and specialized services. This COIL experience enhanced students’ cultural awareness and critical thinking, accentuating the importance of cross-cultural dialogue in addressing global health disparities.

